# Prognostic Value of Single Nodal Zone Metastasis in Non-Small-Cell Lung Cancer—A Multi-Institutional Study

**DOI:** 10.3390/jcm14092938

**Published:** 2025-04-24

**Authors:** Samanta Nicosia, Paraskevas Lyberis, Stefano Rudella, Paolo Olivo Lausi, Simona Sobrero, Riccardo Carlo Cristofori, Matteo Roffinella, Elisa Carla Fontana, Francesco Leo, Enrico Ruffini, Francesco Guerrera

**Affiliations:** 1 Department of Surgical Sciences, University of Torino, Corso Dogliotti, 14, 10126 Torino, Italy; p.lyberis@gmail.com (P.L.); paolo.lausi@unito.it (P.O.L.); enrico.ruffini@unito.it (E.R.); francesco.guerrera@unito.it (F.G.); 2 Department of Thoracic Surgery, AOU Città Della Salute e Della Scienze di Torino, 10126 Torino, Italy; riccardocarlocristofori@gmail.com (R.C.C.); matteo.roffinella@unito.it (M.R.); elisa.fontana88@gmail.com (E.C.F.); 3 AOU San Luigi Gonzaga, Thoracic Surgery Division, Department of Oncology, University of Torino, 10093 Orbassano, Italy; stefano.rudella@unito.it (S.R.); simona.sobrero@unito.it (S.S.); francesco.leo@unito.it (F.L.)

**Keywords:** lung cancer, stage III (N2), locally advanced, lymph node, tailored-therapy, single-zone

## Abstract

**Objectives**: Lung cancer is the leading cause of cancer-related deaths worldwide and mediastinal lymph node involvement is an important negative prognostic factor. Nevertheless, the involvement of a single mediastinal nodal zone has been reported to have favorable outcomes. This study aims to assess whether the prognosis of non-small-cell lung cancer (NSCLC) with single-zone lymph node involvement varies by the affected lymph node zone. **Methods**: We retrospectively analyzed patients affected by NSCLC with a single lymph node zone involvement who underwent anatomical resection. The prognosis of patients was statistically compared based on the different affected lymph node zones. **Results**: A total of 135 patients were enrolled. All patients underwent anatomical lung resection and systematic lymph node dissection. Lymph node involvement was observed in 50 cases (37%) for the upper zone, 36 cases (27%) for the aorto-pulmonary (AP) zone, 41 cases (30%) for the subcarinal zone and 8 cases (6%) for the lower zone. The median follow-up was 37 months [ranging from 1 to 115 months]. Cancer recurrence was reported in 64 cases (52%) during this period. The 2-year and 4-year overall survival (OS) were 69% and 49%, respectively. The 2-year and 4-year relapse-free survival (RFS) were 55% and 41%. The OS and RFS change relating to the different involved lymph node zones (*p* < 0.01). Lower zone involvement predicts worse prognosis, upper zone and subcarinal zone better outcomes, and the AP zone involvement intermediate survival. **Conclusions**: The location of the affected lymph nodes appears to be an important prognostic factor in patients with NSCLC, with significant impacts on both OS and RFS. It may play a key role in the disease progression and patient survival by providing more personalized therapy.

## 1. Introduction

Lung cancer is the second-most diagnosed cancer and remains the leading cause of cancer-related deaths worldwide [1,2]. The most common type of lung cancer is non-small-cell lung cancer (NSCLC), which accounts for over 85% of all diagnosed lung cancers [3]. About one out of three new cases of NSCLC are diagnosed at a locally advanced stage—mainly stage IIIA and IIIB [4], according to the eighth edition of TNM [5]. Stage IIIA and IIIB disease include a widely heterogeneous group of patients presenting significant differences in tumor volume, local diffusion, and lymph node involvement (N). These differences lead to various clinical scenarios with a heterogeneous spectrum of therapeutic options [4]. Common treatment strategies for NSCLC include chemotherapy, the cornerstone of treatment, surgery, and concurrent chemoradiotherapy. Notably, preoperative chemotherapy has significantly improved overall survival (OS) among patients with resectable locally advanced NSCLC [6]. In recent years, immunotherapy has emerged as a transformative treatment strategy for patients with locally advanced NSCLC, ensuring a pathologically complete response and more prolonged survival than chemotherapy alone [7]. This study aims to determine whether the prognosis of patients with NSCLC stage IIIA and IIIB with single-zone lymph node involvement differs depending on the specific lymph node zone involved in the disease. By focusing on single-zone involvement, the study aims to uncover potential differences in outcomes that could guide more tailored treatment strategies and improve overall patient management in this subgroup.

## 2. Materials and Methods

For the study, we enrolled patients referred to Azienda Ospedaliera Universitaria Città della Salute e della Scienza of Torino (Italy), and to Azienda Ospedaliera Universitaria San Luigi Gonzaga of Orbassano (Italy), affected by locally advanced NSCLC, who had undergone anatomical resection and systematic mediastinal lymphadenectomy, with the involvement of a single nodal zone identified pre-operatively and confirmed either through pathological examination of the surgical specimen or detected intraoperatively and subsequently confirmed histologically. All mediastinal tissue containing lymph nodes was dissected and excised within established anatomical landmarks. Dissection of the lymph nodes in the lower zone were always dissected during lower lobectomies and, when identifiable during the pulmonary ligament division, also in upper or middle lobectomies. Upper zone lymphadenectomy was routinely performed during right upper, middle and lower lobectomies. Aortopulmonary zone (AP zone) lymphadenectomy was consistently conducted in all cases of left upper lobectomy. Subcarinal lymph node dissection was uniformly carried out regardless of the type of lobectomy. Written informed written consent for the use of clinical data was obtained from all patients prior to surgery.

Inclusion criteria are as follows:

1. Surgery time ranging from January 2015 to December 2022;
2. NSCLC (adenocarcinoma and squamous cell carcinoma);
3. Operable case with anatomical resection (lobectomy, bilobectomy, pneumonectomy);
4. N2 single zone pathologically confirmed post-surgery.

Exclusion criteria included the following:

1. N2-multizone;
2. Mixed histotype;
3. Presence of metastases at diagnosis;
4. Non-anatomical resection;
5. Salvage surgery.

Pre-operatively, all patients underwent total body computer tomography (TB-CT) and positron emission tomography scans with fluorodeoxyglucose (PET-FDG). Lymph nodes that were >1.5 cm in short diameter in chest CT or showed a maximum standardized uptake value (SUV) > 2.5 in PET-CT were considered metastatic in the clinical staging. Mediastinoscopy and endobronchial ultrasound-guided transbronchial needle aspiration (EBUS-TBNA) was performed for selected patients with suspected N2 at radiological examinations. Surgical resections were performed through postero-lateral thoracotomy incision or video-assisted thoracic surgery (VATS). Anatomical resection (lobectomy, bilobectomy, pneumonectomy) and systematic mediastinal lymphadenectomy were performed in all patients [8].

In conducting our study, we assessed the clinical profile of the patients, including their medical records, laboratory results, radiological examinations, and pathology reports, using our informatic healthcare software (Chromecare, Gesaarc, Architor). Histopathological findings and pathological stage of the disease were described following the guidelines of the International Association for the Study of Lung Cancer (IASLC). Patients were evaluated every 4 or 6 months through ambulatory visits or phone contact. Associations between clinicopathological characteristics and the lymph node zone involved by the tumor were investigated using analysis of variance (ANOVA) for continuous variables and the Fisher’s exact test for categorical variables.

The OS and relapse-free survival (RFS) were estimated by the Kaplan–Meier method. The observation period for OS was defined as the time from the date of surgery to the date of death by any cause (event) or to the date of the last follow-up visit (censoring). The observation period for RFS was defined as the time from the date of surgery to the date of tumor recurrence (event) or to the date of the last follow-up visit or death for any cause (censoring). A Cox proportional hazard model was employed to estimate the crude and the multivariable-adjusted hazard ratios (HRs) with 95% confidence intervals (CIs) and evaluate possible OS and PFS predictors. Graphical checks and formal tests based on Schoenfeld residuals also verified the proportional hazard assumption. To find the best multivariable-adjusted model for each outcome, a stepwise backward selection was used. To handle missing data, a listwise deletion approach (i.e., complete case analysis) was used. Multicollinearity between the selected covariates in the multivariable analysis was assessed by testing the variance inflation factor. Statistical analyses were conducted using Stata software version 18 (Stata—Corp, College Station, TX, USA).

## 3. Results

For the study, we enrolled a total of 135 patients: 82 male (61%) and 53 female (39%). Table 1 and Table 2 summarize the clinicopathological features of the study population.

The mean age was 68 years [ranging from 37 to 85]. One hundred fifteen patients (85%) were active smokers or ex-smokers, and 20 patients (15%) had never smoked. One hundred thirteen patients (84%) underwent postero-lateral thoracotomy, 21 patients (16%) underwent VATS, and one patient (<1%) underwent robotic-assisted thoracic surgery (RATS). In the recruited cases, 23 cases (17%) were identified as squamous cell carcinomas (SCCs), while 112 cases (83%) as adenocarcinoma (ADCs). According to the eighth edition of TNM, 88 patients (65%) were in stage IIIA, while 47 patients (35%) were in stage IIIB. According to the ninth edition of TNM, 32 (24%) patients were in stage IIB, 71 patients (52%) were in stage IIIA, and 32 (24%) patients were in stage IIIB. Lobectomy was performed in 117 patients (87%), bilobectomy in 6 patients (<1%), and pneumonectomy in 17 patients (13%). Exactly 28 patients (21%) were cN2, and only 12 of them received neoadjuvant chemotherapy (43%), while 107 patients (79%) were occult N2. Seventy-four patients (60%) received planned postoperative adjuvant cisplatin-based chemotherapy. Forty-one patients (37%) received adjuvant mediastinal radiotherapy (54–60 Gy); among these, 9 patients received only radiotherapy and 32 received radiotherapy plus chemotherapy. Thirteen patients (<1%) received adjuvant immunotherapy (pembrolizumab or osimertinib or nivolumab). Sixty-four patients (47%) experienced disease progression, 75 patients (55%) died during the follow-up period.

**Figure 1:**
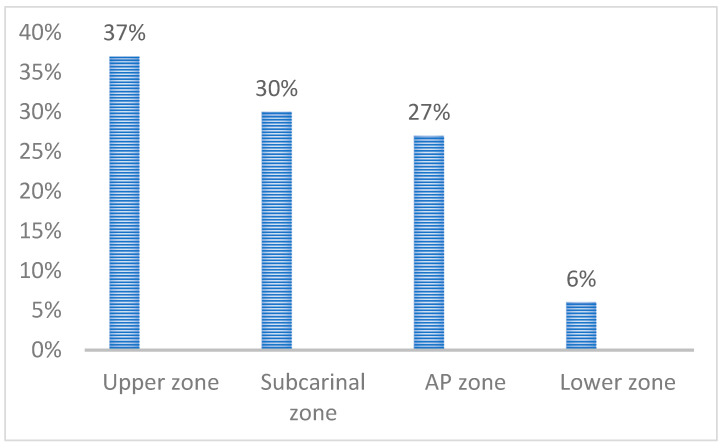
Lymph node zone involvement.

The lymph node involvement of the upper zone occurred in 50 cases (37%); the involvement of the aortopulmonary zone (AP zone) in 36 cases (27%), involvement of the subcarinal zone in 41 cases (30%), and involvement of the lower zone lymph node in 8 cases (6%), as shown in Figure 1 and Table 3.

### 3.1. Overall Survival Analysis

**Figure 2:**
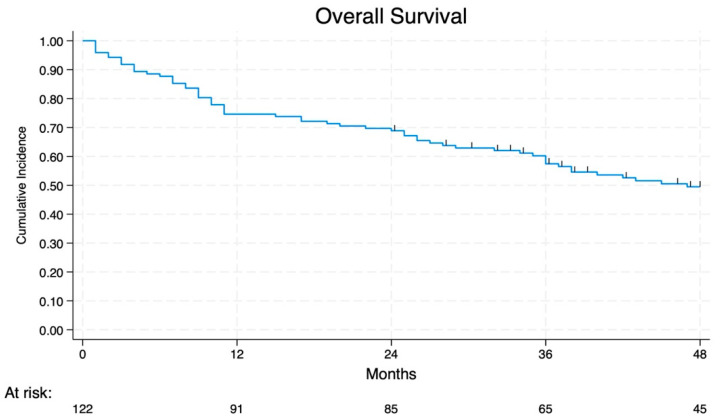
Overall survival of all groups.

**Figure 3:**
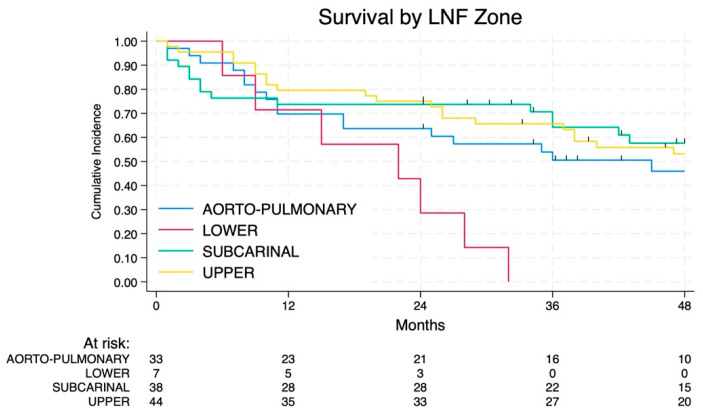
Survival by lymph node zone.

**Figure 4:**
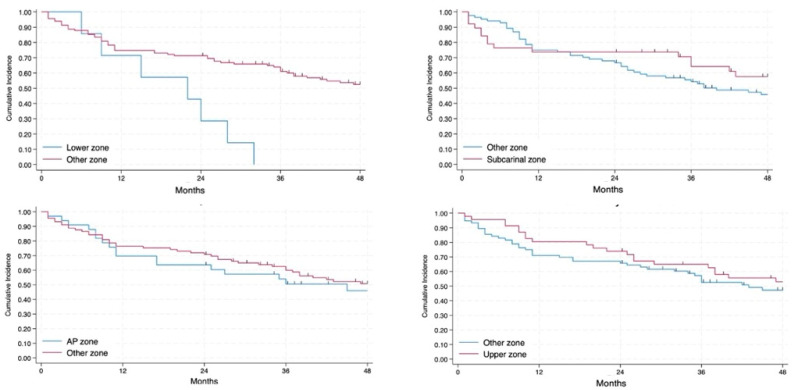
Involvement of each lymph node zone in relation to the others in terms of OS.

The 2-year and 4-year OS were 69% and 49%, respectively (Figure 2). The OS of patients affected by NSCLC with N2 disease changed in relation to the different lymph node zones involved (*p* < 0.01), as shown in Figure 3 and Figure 4. In particular, the involvement of the lower zone is associated with the worst prognosis (*p* < 0.01).

On univariable analysis, the involvement of the upper zone (*p*: 0.006, 95% CI 0.124–0.701), subcarinal zone (*p*: 0.002, 95% CI 0.095–0.588) and AP zone (*p*: 0.016, 95% CI 0.138–0.818) are significantly associated with an improved OS than involvement of the lower zone (Table 4).

On the multivariable model (adjusted for age, sex, gender, smoking, CCI, stage, and perioperative therapy, Table 3), the involvement of the subcarinal zone (*p* < 0.001, 95% CI 0.040–0.339), upper zone (*p*: 0.001, 95% CI 0.075–0.512) and AP zone (*p*: 0.021, 95% CI 0.125–0.845) seems to have a protective effect.

### 3.2. Relapse-Free Survival Analysis

**Figure 5:**
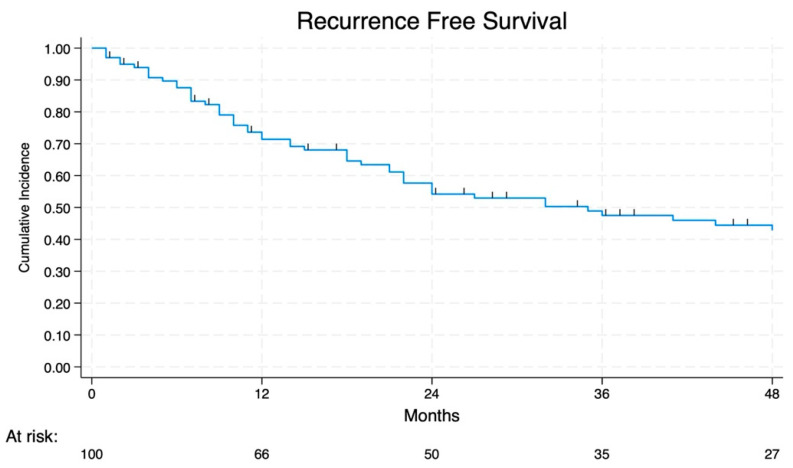
RFS of all groups.

During the follow-up period, cancer recurrence was reported in 33 cases (49%). The recurrence sites were primarily locoregional, such as the brain, adrenal gland, liver, and bones. The treatment of recurrence was radiotherapy, chemotherapy, or immunotherapy. Over time, the probability of having a recurrence progressively increases. The 2-year and 4-year RFS rates were 55% and 41%, respectively (Figure 5).

**Figure 6:**
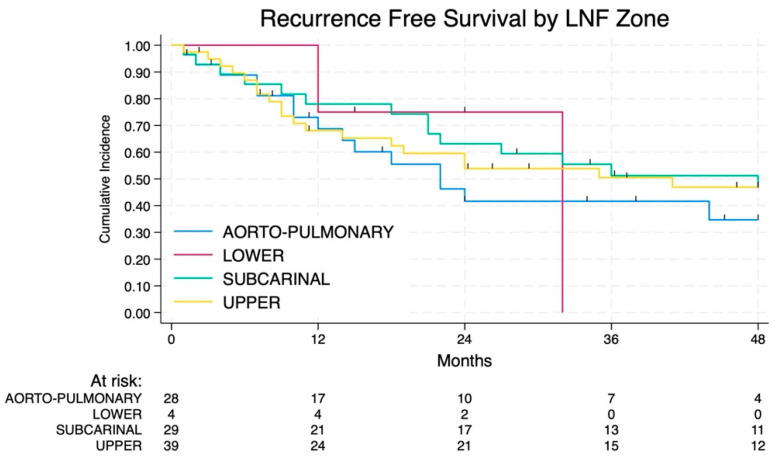
RFS according to the different lymph node zone involved.

**Figure 7:**
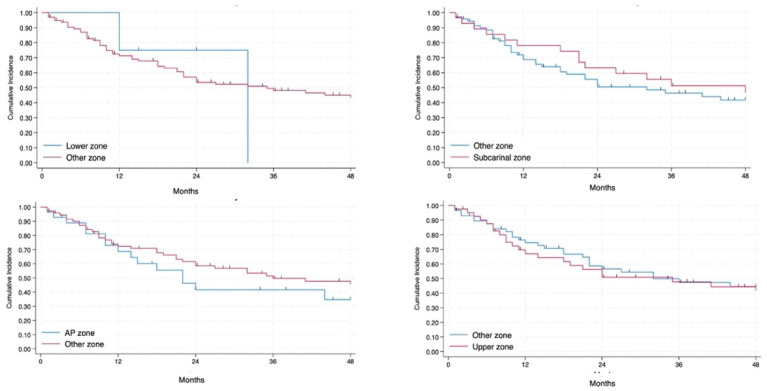
Involvement of each lymph node zone with regard to the others in terms of RFS.

The involved lymph node zone appears to have a different impact on RFS without statistical significance (Figure 6 and Figure 7): patients with the involvement of the lower zone and AP zone had the worst prognosis whereas involvement of subcarinal and upper zone is associated with a more favorable prognosis.

## 4. Discussion

NSCLC with N2 single-zone involvement represents a highly heterogeneous group of lung tumors with significant differences in tumor size, local infiltration, and lymph nodal involvement. In this group of patients, therapy is based on the pivotal role of multimodal treatments, including surgery, a wide-ranging option of systemic therapies, radiotherapy, and immunotherapy. The selection of these various strategies is inherently limited by patients’ comorbidities and clinical staging, particularly when it differs from the pathological stage (e.g., in cases of occult N2), potentially introducing bias into the study. When stage III NSCLC is considered potentially resectable, the goal of the multimodal treatment is curative [4]. After discussion within the multidisciplinary team, patients should become candidates for induction systemic treatment or proceed directly to surgery.

Lymphadenectomy is a critical component of surgical treatment for NSCLC. One of the most critical issues in thoracic oncology is the accurate description of metastasis to locoregional lymph node stations. N2 lymph node involvement is one of the most important prognostic factors in patients with NSCLC: it plays an essential role in the therapeutic and postoperative staging of lung cancer. Given its impact on staging accuracy, treatment decisions, surgical planning, and patient outcomes, systematic nodal dissection has become the standard of care in the surgical management of NSCLC [8]. Without this thorough approach, there is a risk of under-staging, leading to inadequate treatment and worse outcomes.

A recent nationwide, retrospective, multicentre cohort study [9] aimed to identify the factors influencing the quality of lymphadenectomy by analyzing data from patients who underwent minimally invasive surgery for early-stage NSCLC. The results showed that patients who underwent preoperative PET-CT, those with larger tumors, and those operated on by experienced surgeons had a higher likelihood of adequate lymphadenectomy. Notably, the analysis revealed a concerning decline in lymphadenectomy quality over time. However, a more extensive lymph node dissection was not associated with higher complication rates or in-hospital mortality. These findings highlight the need for interventions to improve the quality of lymphadenectomy in lung cancer surgery.

It is essential to designate each nodal station accurately for an exact nodal assessment. Determining the exact nodal station can be challenging or unclear due to the continuity between adjacent nodal stations, especially in the case of subcarinal lymph nodes and hilar lymph nodes or upper paratracheal nodes and lower paratracheal nodes. The concept of a nodal zone was proposed to solve these matters partially. The IASLC proposed a lymph node zone staging project in 2009 [10,11]. Nodal stations are grouped into seven zones. So, a nodal zone is an anatomical area that includes one or several neighboring nodal stations. The upper zone includes the lymph nodes located above the tracheal bifurcation, specifically the high and low paratracheal lymph nodes (station 2R, 2L, 4R, and 4L). The lower zone includes paraesophageal lymph nodes and lymph nodes of the pulmonary ligament (station 8 and 9). The subcarinal zone includes the subcarinal lymph nodes (station 7). The aortopulmonary (AP) zone includes subaortic and para-aortic lymph nodes (station 5 and 6). The concept of the nodal zone has changed how locally advanced NSCLC is approached.

The literature showed no statistically significant survival differences between the single-station and multiple-station metastases groups when only one nodal zone was involved [12]. However, research has demonstrated that 5-year survival was significantly better for patients with single-nodal-zone involvement compared with multizone; in addition, patients with single-zone nodal involvement, even if multistation is included, have a survival outcome like that of patients with single-station nodal involvement [12]. Although mediastinal metastasis in NSCLC is related to poor prognosis, single mediastinal nodal station metastasis in surgically resected NSCLC patients has been reported to have favorable outcomes.

The most recent TNM classification for NSCLC is the ninth edition [13,14], implemented in clinical practice from 1st January 2025. This updated classification reflects advances in understanding lung cancer staging and aims to provide more accurate and relevant staging criteria focusing on lymph node involvement. These changes are intended to enhance the accuracy of prognosis, guide treatment decisions more effectively, and ultimately improve patient outcomes. The ninth edition of the TNM allows the quantification of node disease by dividing the N2 category into N2a and N2b subcategories [14]. The prognostic relevance of quantifying nodal disease was evident in the previous two editions of the TNM classification, either by the number of involved nodal zones or by the number of involved nodal stations: the greater the number of involved nodal zones or nodal stations, respectively, the worse the prognosis.

The ninth edition of the TNM classification focuses on the number of lymph node stations involved rather than their location. In this study, we focus our attention on the location of mediastinal lymph nodes involved by the tumor to determine whether it is a parameter influencing the prognosis.

According to the current literature, it is unclear whether the specific lymph node zone involved affects the prognosis of NSCLC. There are some disagreements about the effects of subcarinal nodal station involvement on survival; some authors have argued that the presence of subcarinal nodal metastases is associated with a poor prognosis, and others have argued that survival is not significantly influenced by subcarinal node metastasis [12]. In our study, the impact of subcarinal lymph node involvement seems to have a positive effect in term of OS (*p* < 0.001 in multivariable analyses) and intermediate in term of RFS. This can be justified by the fact that the subcarinal lymph node has a central position within the thoracic lymphatic drainage system, and its involvement might be associated with a robust immune response to the tumor. Another possible explanation could be that the involvement of subcarinal lymph nodes is more easily detected during the pre-operative phase, which could lead to more aggressive treatments that may indirectly contribute to a better outcome in this subgroup of patients.

Some research reported that patients with the involvement of lymph nodes from the AP zone, treated with left upper lobectomy with curative intent, have better survival than those of other zones [15]. According to our study, the OS of patients with involvement of the AP lymph node zone shows a negative trend with a progressive reduction over time, but it is less pronounced compared to the rapid decline observed for the lower zone. In term of RFS, the involvement of the AP zone is associated with poorer outcomes, characterized by a rapid decline within the first 24 months.

The results of our study suggest that lower zone lymph node involvement in patients with NSCLC is significantly associated with reduced OS and RFS thus representing an adverse prognostic indicator. Moreover, lower zone lymph nodes are notoriously difficult to investigate preoperatively, representing a significant challenge in accurately staging the tumor and planning an optimal upfront diagnostic-staging pathway. Given these limitations, patients with lower zone involvement require more aggressive and carefully tailored management, discussed in a multidisciplinary team. However, therapeutic options may be restricted due to the location of the affected lymph nodes and the potential complications associated with treatments such as radiotherapy or the often-poor response to chemotherapy.

Involvement of the upper zone appears more favorable in terms of OS (*p*: 0.001 in multivariable analyses) and RFS. This could be attributed to a more straightforward and complete surgical resection, leading to a lower probability of recurrence, or to an improved response and greater effectiveness of multimodal treatments.

Although OS and RFS differ significantly among groups based on lymph node zone involvement, the analysis of clinical and pathological features (gender, age, smoking status, CCI, cN2 status, occult N2, TNM stage, and pre- or postoperative treatments) revealed no statistically significant differences between the groups. This homogeneity suggests that the observed prognostic differences are unlikely to be attributable to confounding clinical factors. Instead, these findings support the hypothesis that the anatomical location of lymph node involvement may represent an independent prognostic factor, not mediated by traditionally considered clinical variables.

The limitations of our study should be acknowledged. First, this was a retrospective study, which inherently carries the risk of various biases that may not have been identified and controlled. Secondly, the study population is relatively small, which may reduce the statistical power to detect a significant difference between OS and RFS. In particular, the subgroup of patients with involvement of the lower zone (n = 8) is notably small. This may be attributed to the patient selection process: the inclusion criteria could have further reduced the number of cases with the involvement of this zone, as the lower zone is difficult to assess with pre-operative exams, such that all these patients were cN0 (occult N2). Additionally, involvement of the lower zone is often associated with involvement of other nodal regions, and multi-zone N2 disease is an exclusion criterion for the study. Consequently, these factors could potentially lead to a statistical artifact rather than representing a true prognostic factor. Third, pre-operative mediastinoscopy and EBUS were not routinely performed as clinical staging of suspicious nodes before resection; this could be a source of bias, as some patients with a poor prognosis may be included in this study. Finally, the study period was relatively long (six years): significant advancements in preoperative diagnostic protocols and perioperative and postoperative techniques could have influenced the prognosis.

It is crucial to carefully consider the surgical strategy adopted for each patient. First, it is important to highlight the combination of different surgical approaches (traditional surgery, VATS, and RATS). While the surgical access itself does not directly affect OS, it is plausible that patients undergoing resection via minimally invasive surgery had smaller tumors compared to those undergoing traditional surgery. Furthermore, patients treated using a minimally invasive approach may have benefited from a more precise and complete lymph node dissection, leading to more accurate tumor staging and, consequently, a better prognosis. Additionally, the inclusion of patients undergoing both lobectomy and pneumonectomy represents another potential limitation. Pneumonectomy patients typically have larger tumors, less favorable tumor locations, and require more extensive resections, which could result in a less favorable prognosis compared to patients undergoing lobectomy. Combining these two groups in a single analysis could distort the results, as pneumonectomy patients may have lower OS due to tumor characteristics and the greater complexity of the surgical procedure.

To improve the validity of the study, it may be advisable to stratify patients into more homogeneous groups thereby reducing the risk of bias and ensuring that conclusions are as accurate and representative as possible.

## 5. Conclusions

The localization of affected lymph nodes is not only a useful clinical parameter for staging, but also a true prognostic factor that guides the therapeutic strategy. Although the statistical power is weak because of the small population, it clearly emerges that the site of lymph node involvement profoundly influences the course of the disease in patients with NSCLC, with repercussions both on OS and RFS.

The results highlight the importance of considering the anatomical localization of N2 disease as a potentially relevant prognostic factor. Consequently, incorporating the specific lymph node zone involved into therapeutic decision-making, risk stratification, and the design of future studies may be beneficial. This approach could enhance clinical decision-making and contribute to more personalized and effective treatment strategies, maximizing the chances of success while minimizing potential side effects.

This investigation could drive future research into how lymph node location affects disease progression and response to treatment. It could also contribute to the development of new guidelines or modifications to existing staging systems.

Confirming that the involvement of the lower zone is a negative prognostic factor could lead to the necessity and recommendation of a systematic dissection of this lymph node zone in all cases of lung cancer. If studies consistently demonstrate that lower zone involvement is associated with worse outcomes, it could justify a more aggressive surgical approach to ensure more complete tumor staging and potentially enhance survival rates by enabling a more tailored and effective adjuvant therapy.

Further prospective, randomized controlled trials with a similar background but with a larger cohort are necessary to validate these preliminary results and provide more robust conclusions.

## Figures and Tables

**Table 1:** Clinicopathological characteristics of 135 patients with N2—single zone NSCLC, expressed in absolute numbers and in percentages.

	All
Patients—*n* (%)	135
Age—mean (SD)	68 (9)
Gender—*n* (%)	
Male	82 (61)
Female	53 (39)
Smoker—*n* (%)	
Yes	115 (85)
No	20 (15)
Charlson Comorbidity Index (CCI)—mean (SD)	5 (2)
cN2—*n* (%)	28 (21)
Occult N2—*n* (%)	107 (79)
TNM 8 ED.—*n* (%)	
IIIA	88 (65)
IIIB	47 (35)
TNM 9. ED.—*n* (%)	
IIIA	71 (52)
IIIB	32 (24)
II B	32 (24)
Neoadjuvant—*n* (%)	12 (<1)
Adjuvant—*n* (%)	74 (60)

**Table 2:** Clinicopathological characteristics distribution by lymph node area, expressed in absolute numbers and in percentages.

Characteristics	Upper Zone	Ap Zone	Subcarinal Zone	Lower Zone	*p*
Patients—*n* (%)	50 (37)	36 (27)	41 (30)	8 (6)	
Age—mean (SD)	67 (9.4)	68.6 (8.4)	66.6 (8.9)	67.6 (6.7)	0.778
Gender—*n* (%)					0.165
Male	30 (60)	20 (55)	29 (71)	5 (62)	
Female	20 (40)	16 (44)	12 (29)	3 (38)	
Smoker—*n* (%)					0.293
Yes	42 (84)	28 (77)	38 (93)	7 (88)	
No	8 (16)	8 (23)	3 (7)	1 (12)	
CCI—mean (SD)	5 (2)	6 (2)	6 (1)	5 (1)	0.618
cN2—*n* (%)	15 (30)	8 (23)	4 (10)	1 (12)	0.111
Occult N2—*n* (%)	35 (70)	28 (77)	37 (90)	7 (88)	0.112
TNM 8 ED.—*n* (%)					0.722
IIIA	31 (62)	21 (58)	29 (71)	5 (62)	
IIIB	19 (38)	15 (4)2	12 (29)	3 (38)	
TNM 9. ED.—*n* (%)					0.841
IIIA	23 (46)	20 (55)	23 (56)	4 (50)	
IIIB	12 (24)	10 (28)	8 (20)	3 (38)	
II B	15 (30)	6 (17)	10 (24)	1 (12)	
Neoadjuvant—*n* (%)	7 (14)	3 (8)	1 (2)	1 (12)	0.176
Adjuvant—*n* (%)	24 (48)	23 (64)	25 (61)	2 (25)	0.496

**Table 3:** Lung resection and respective involved lymph node zone (data are reported in absolute numbers and in percentages).

	RUL *n* (%)	ML *n* (%)	RLL *n* (%)	LUL *n* (%)	LLL *n* (%)	Right Pneumo *n* (%)	Left Pneumo *n* (%)	Bilobectomy *n* (%)	TOT *n* (%)
Upper zone	40 (30)	0	5 (4)	0	1 (<1)	3 (2)	0	1 (<1)	50 (37)
AP zone	0	0	0	25 (19)	3 (2)	0	8 (6)	0	36 (27)
Subcarinal zone	3(2)	2 (1)	23 (17)	1 (<1)	2 (1)	2 (1)	4 (3	4 (3)	41 (30)
Lower zone	2 (1)	0	2 (1)	0	3 (2)	0	0	1 (<1)	8 (6)
TOT	45 (33)	2 (1)	30 (22)	26 (19)	9 (7)	5 (4)	12 (9)	6 (4)	135

**Table 4:** Univariable and multivariable analyses for OS; * the variables used in the analyses are adjusted for age, sex, gender, smoking, CCI, stage, and perioperative therapy.

Variable *	HR	*p* Value	95% Conf. Interval	
			Lower	Upper
Univariate				
Subcarinal zone	0.2367979	0.002	0.095	0.588
AP zone	0.3366773	0.016	0.138	0.818
Upper zone	0.2956162	0.006	0.124	0.7011
Multivariate				
Subcarinal Zone	0.1174965	<0.001	0.040	0.339
AP zone	0.3263782	0.021	0.125	0.845
Upper zone	0.1968624	0.001	0.075	0.512

## Data Availability

The data presented in this study are available on request from the corresponding author.

## Abbreviations

The following abbreviations are used in this manuscript:

IASLC	International Association for the Study of Lung Cancer
OS	Overall survival
RFS	Relapse-free survival
AP zone	Aortopulmonary zone
